# Supplementary material for: Computer tomography guided thoracoscopic resection of small pulmonary nodules in the hybrid theatre
Source: PLoS One. 2021 Nov 3;16(11):e0258896. doi: 10.1371/journal.pone.0258896 (PMC8565725; doi:10.1371/journal.pone.0258896)
Supplement: S1 File — (DOCX) [file pone.0258896.s002.docx]

Supplementary Material

**Table 2: Patient characteristics (n= 50 patients)**

| N | Sex | suspected diagnosis | final diagnosis | Result |
| --- | --- | --- | --- | --- |
| 1 | F | Malignant Melanoma | Malignant Melanoma | Confirmed |
| 2 | F | Anal cancer (M1) | NSCLC | NSCLC: AIS |
| 3 | F | HCC | HCC | Confirmed |
| 4 | F | Synovial Sarcoma | Synovial Sarcoma | Confirmed |
| 5 | F | CRC | NSCLC in situ | NSCLC: AIS |
| 6 | M | Malignant Melanoma | Malignant Melanoma | Confirmed |
| 7 | F | GGO | NSCLC | NSCLC: invasive adenocarcinoma |
| 8 | M | Prostate Ca. | Prostate Ca. | Confirmed |
| 9 | M | Oropharynx Ca. | Granuloma | Benign disease |
| 10 | M | RCC | RCC | Confirmed |
| 11 | F | GGO | NSCLC | NSCLC: invasive adenocarcinoma |
| 12 | F | RCC | RCC | Confirmed |
| 13 | F | NSCLC | Lymphnode | Benign disease |
| 14 | F | Malignant Melanoma | Lymphnodes | Benign disease |
| 15 | M | Malignant Melanoma | Tuberculosis | Other disease requiring treatment |
| 16 | M | Malignant Melanoma | Lymphnode | Benign disease |
| 17 | F | Transitional cell Ca. | Transitional cell Ca. | Confirmed |
| 18 | F | NSCLC | NSCLC | Confirmed |
| 19 | M | RCC | RCC | Confirmed |
| 20 | M | CRC | CRC | Confirmed |
| 21 | M | CRC | Lymphnode | Benign disease |
| 22 | F | CRC | CRC | Confirmed |
| 23 | M | Osteosarcoma | Osteosarcoma | Confirmed |
| 24 | M | RCC | RCC | Confirmed |
| 25 | F | CRC | CRC | Confirmed |
| 26 | F | CRC | Hamartoma | Benign disease |
| 27 | M | NSCLC | De novo NSCLC | NSCLC: invasive adenocarcinoma |
| 28 | M | CRC | CRC | Confirmed |
| 29 | M | Malignant Melanoma | Malignant Melanoma | Confirmed |
| 30 | M | Leiomyosarcoma | Leiomyosarcoma | Confirmed |
| 31 | M | CRC | CRC | Confirmed |
| 32 | F | RCC | RCC | Confirmed |
| 33 | M | Prostate Ca | NSCLC | NSCLC: invasive adenocarcinoma |
| 34 | M | RCC | RCC | Confirmed |
| 35 | M | Malignant Melanoma | Malignant Melanoma | Confirmed |
| 36 | M | GIST | Tuberculosis | Other disease requiring treatment |
| 37 | F | Cervix Ca. | NSCLC | NSCLC: invasive adenocarcinoma |
| 38 | M | Urothelial Ca. | Inflammation | Benign disease |
| 39 | F | Malignant Melanoma Oropharynx Ca. | NSCLC | NSCLC: invasive SCC |
| 40 | F | CRC | Atelectasis | Benign disease |
| 41 | M | Malignant Melanoma | Malignant Melanoma | Confirmed |
| 42 | F | NSCLC | NSCLC | Confirmed |
| 43 | M | Malignant Melanoma | Malignant Melanoma | Confirmed |
| 44 | M | NSCLC | NSCLC | Confirmed |
| 45 | M | Hypopharynx Ca. | Hypopharynx Ca. | Confirmed |
| 46 | F | GGO | NSCLC | NSCLC: MIA |
| 47 | M | CRC | Hamartoma | Benign disease |
| 48 | F | Parotid cancer Ca. | Both parotis Ca. | Confirmed |
| 49 | M | GGO | Tuberculosis | Other disease requiring treatment |
| 50 | M | CCC | CCC | Confirmed |
| NSCLC: non-small cell lung cancer; HCC: hepatocellular carcinoma; GGO: ground glass opacity; RCC: renal cell carcinoma; Ca.: cancer; CRC: colorectal cancer; GIST: gastrointestinal stromal tumour; CCC: cholangiocellular carcinoma; AIS: adenocarcinoma in situ; MIA: minimally invasive adenocarcinoma; SCC: squamous cell carcinoma | | | | |

**Table 3: CATS procedure (n= 52 lesions)**

| N | Location | Path length (mm) | Lesion diameter (mm) | Lesion depth (mm) |
| --- | --- | --- | --- | --- |
| 1 | RLL | 54,6 | 5,6 | 9 |
| 2 | RLL | 52,7 | 9,4 | 15 |
| 3 | LLL | 56,3 | 10,7 | 11 |
| 4 | LLL | 70,9 | 8,9 | 18 |
| 5 | RUL | n.a. | 10 | 4 |
| 6 | RLL | 74,4 | 8,2 | 17 |
| 7 | RML | 63,5 | 9,2 | 32 |
| 8 | RLL | 80,4 | 7,29 | 12 |
| 9 | LUL | 46,2 | 9 | 13 |
| 10 | RUL | 82 | 8 | 16 |
| 11 | RUL | 83 | 12 | 13 |
| 12 | RUL | 85 | 4,4 | 9 |
| 13 | RLL | 39 | 5 | 16 |
| 14 | LLL | 53 | 6 | 24 |
| 15 | LUL | 53 | 4 | 14 |
| 16 | LLL | 59,5 | 5 | 19 |
| 17 | RML | 86 | 6 | 15 |
| 18 | RLL | 33 | 8 | 11 |
| 19 | LUL | 57 | 6 | 7 |
| 20 | LUL | 65 | 11 | 18 |
| 21 | RUL | 73 | 6 | 8 |
| 22 | LLL | 57 | 6 | 21 |
| 23 | LUL | 37 | 4 | 6 |
| 24 | LLL | 83 | 13 | 36 |
| 25 | LLL | 47 | 9 | 12 |
| 26 | LLL | 51 | 13 | 43 |
| 27 | RML | 49 | 4 | 9 |
| 28 | LLL | 57 | 12 | 10 |
| 29 | LLL | 48 | 7 | 23 |
| 30 | RML | 42 | 7 | 20 |
| 31 | RML | 42 | 5 | 15 |
| 32 | LLL | 68 | 8 | 41 |
| 33 | LLL | 59 | 6 | 17 |
| 34 | RUL | 73 | 12 | 20 |
| 35 | LUL | 91 | 8 | 25 |
| 36 | RML | 52 | 9 | 25 |
| 37 | RUL | 47 | 8 | 30 |
| 38 | RUL | 64 | 7 | 53 |
| 39 | RUL | 68 | 8 | 31 |
| 40 | LLL | 38 | 9 | 13 |
| 41 | LLL | 6 | 17 | 18 |
| 42 | RLL | 75 | 31 | 13 |
| 43 | LUL | 60 | 9 | 35 |
| 44 | RUL | 51 | 3 | 12 |
| 45 | RUL | 60 | 9 | 25 |
| 46 | LLL | 29 | 8 | 9 |
| 47 | RUL | 57 | 12 | 5 |
| 48 | LLL | 60 | 10 | 25 |
| 49 | LLL | 5 | 5 | 23 |
| 50 | LLL | 4 | 5 | 20 |
| 51 | RLL | 60 | 10 | 9 |
| 52 | RLL | 24 | 4 | 7 |
| RUL: right upper lobe; RML: right middle lobe; RLL: right lower lobe; LUL: left upper lobe; LLL: left lower lobe | | | | |
