# Supplementary material for: Computer tomography guided thoracoscopic resection of small pulmonary nodules in the hybrid theatre
Source: PLoS One. 2021 Nov 3;16(11):e0258896. doi: 10.1371/journal.pone.0258896 (PMC8565725; doi:10.1371/journal.pone.0258896)
Supplement: Attachment — Submitted filename: Response to Reviewers (copy).docx [file pone.0258896.s003.docx]

Response to reviewers’ comments:

Dear colleagues, thank you for reviewing our work and for the points you raised. We hope the changes made will meet your expectations.

Response to reviewer 1:

Point 1: Concerning the depth and the location of the nodules, we have added a table as a supplement, where you can find the information that you requested.

Point 2: The maximum depth that you can reach with this technique is probably not a question that we can directly answer. The deepest nodule in this study was a 7 mm lesion lying 53 mm deep in the lung tissue. The path length for the marking wire was 64 mm. However, it is probably the vascular and bronchial anatomy that limits the resection of these nodules and less the absolute depth of the lesion itself, as you can usually manage to place a stapler around the lesion and resect it, if you manage to locate it securely (in rare cases you can run an additional CT scan to make sure that the tip of the wire is enclosed in your stapler).

Point 3: There were 12 patients with benign disease but 13 benign lesions (one patient had two suspicious lesions and both of them turned to be anthracotic lymphnodes, patient #14).

Point 4: Regarding the benign histology of the resected lesions, you are absolutely right. In this study we found a benign pathology in 25% of the cases, which is clearly high. However, the patient population of this study is different from the populations presented in all the well-known trials of patients with incidentally diagnosed solitary nodules or patients undergoing screening for lung cancer. The patients discussed here are not healthy individuals but patients with another known solid tumor and suspected pulmonary metastasis (46 the 50 cases). It is certainly a difficult decision to offer surgery to such a patient. From our point of view and taking the very low complication rate into account, it is rather positive to be able to relieve 25% of these patients from the diagnosis of metastatic cancer.

Point 5: You requested to summarize table 1 and figure 1 according to disease categories. Table 1 has been summarized. The bigger table from the first submission was kept as suppl. material. Figure 1 presents the time that was required for the procedure in each patient. We are not sure, what exactly you mean by summarizing this table based on the histology, as the histology does not really affect the time required to perform the procedure according to our opinion. If you wish to see another figure please explain in detail exactly what type of figure you expect.

Response to reviewer 2:

Point 1: Thank you for the issue you raised. As you pointed out, this is a single institution, retrospective study and not an interim analysis of data that were collected parallel to a formal prospective trial. It is therefore not possible for us to include a CONSORT diagram because the necessary data on enrollment, eligibility etc. are not available.

Point 2: Another table has been created and submitted as suppl. material including the information that you requested. We have changed the medians with means.

Point 3: The changes you requested have been made accordingly and are included in the table in the suppl. material.

Pont 4: This is an excellent comment. It is our policy not to extend surgery in this specific subgroup of patients (patient with a malignant background undergoing operation for suspected metastasis) directly to an oncological resection for two different reasons: first, the result of the frozen section that we receive does not always allow us to securely differentiate metastatic disease from a new primary tumour and we often have to wait for the immunohistochemistry. Second, these patients have not received formal staging for lung cancer according to the national guidelines so we might end up performing a lobectomy in a patient with extensive disease, which is not justified. We prefer to complete the staging and then discuss with the patient the possibility of a completion lobectomy with SND, follow up or other types of treatment, depending on the histology and stage of the disease. Our policy is different for patient without previous malignant disease.

Point 5: The treatment changes have been added in table 1

Point 6 + 7: The purpose of this article was not to describe CATS as a method but to discuss the histopathological findings of this specific patient cohort, that was operated using this method. This is the reason why we did not go into further detail in the CATS complications or discussed this method compared with other localization techniques. However, due to the fact that the latter was pointed out from both the reviewers and the editor we have added a paragraph in the discussion focused on this point.

Point 8: Thank you for pointing this out, we have cross-read the article again for typing errors and hope we have not missed further errors.
